# Estimating Site Performance (ESP): can trial managers predict recruitment success at trial sites? An exploratory study

**DOI:** 10.1186/s13063-019-3287-6

**Published:** 2019-04-03

**Authors:** Hanne Bruhn, Shaun Treweek, Anne Duncan, Kirsty Shearer, Sarah Cameron, Karen Campbell, Karen Innes, Dawn McRae, Seonaidh C. Cotton

**Affiliations:** 1 Health Services Research Unit, University of Aberdeen, Health Sciences Building, Foresterhill, Aberdeen, UK; 2 CHaRT, University of Aberdeen, Health Sciences Building, Foresterhill, Aberdeen, UK; 3 NHS Grampian, Aberdeen Royal Infirmary, Foresterhill, Aberdeen, UK

**Keywords:** Recruitment, Clinical trials, Trial managers, Recruitment sites, Recruitment performance

## Abstract

**Background:**

Multicentre randomised trials provide some of the key evidence underpinning healthcare practice around the world. They are also hard work and generally expensive. Some of this work and expense are devoted to sites that fail to recruit as many participants as expected. Methods to identify sites that will recruit to target would be helpful.

**Methods:**

We asked trial managers at the Centre for Healthcare Randomised Trials (CHaRT), University of Aberdeen to predict whether a site would recruit to target. Predictions were made after a site initiation visit and were collected on a form comprising a simple ‘Yes/No’ prediction and a reason for the prediction. We did not provide guidance as to what trial managers might want to think about when making predictions.

After a minimum of eight months of recruitment at each site for which a prediction had been made, all trial mangers in CHaRT were invited to a group discussion where predictions were presented together with sites’ actual recruitment performance over that period. Individual trial managers reflected on their predictions and there was a general discussion about predicting site recruitment. The prediction reasons from the forms and the content of the group discussion were used to identify features linked to correct predictions of recruitment failure.

**Results:**

Ten trial managers made predictions for 56 site visits recruiting to eight trials. Trial managers’ sensitivity was 82% and their specificity was 32%, correctly identifying 65% of sites that would hit their recruitment target and 54% of those that did not. Eight ‘red flags’ for recruitment failure were identified: previous poor site performance; slow approvals process; strong staff/patient preferences; the site recruitment target; the trial protocol and its implementation at the site; lack of staff engagement; lack of research experience among site staff; and busy site staff. We used these red flags to develop a guided prediction form.

**Conclusions:**

Trial managers’ unguided recruitment predictions were not bad but were not good enough for decision-making. We have developed a modified prediction form that includes eight flags to consider before making a prediction. We encourage anyone interested in contributing to its evaluation to contact us.

**Electronic supplementary material:**

The online version of this article (10.1186/s13063-019-3287-6) contains supplementary material, which is available to authorized users.

## Introduction

Multicentre randomised trials provide some of the key evidence underpinning healthcare practice around the world. They are also hard work and generally expensive.

Some of this work and expense is devoted to sites that fail to recruit as many participants as expected or simply fail to recruit at all. This contributes to the well-known fact that many trials struggle to recruit participants. For the UK National Institute of Health Research Health Technology Assessment programme, a major public funder of multicentre trials in the UK, around half of all trials fail to recruit to target [1–3]. In the UK and elsewhere, many trials are abandoned; a Swiss study of > 1000 trials (two-thirds sponsored by industry) found that 25% were abandoned, chiefly because of recruitment problems, administrative issues and running out of money [4]. A US study of one academic medical centre found 260 trials abandoned because of poor recruitment over a five-year period at a cost of almost $1 million [5]. Even trials that meet their overall recruitment target can have large recruitment variations across sites. Recruitment across the 13 sites in UKCTOCS ranged from 19% of those eligible to 33% [6]. In BeWEL, 98% of participants came from just three of the five sites, despite the substantial amount of time spent by the central trial team on the two other sites [7]. This contributes to research waste [8] and is one reason why recruitment is the top research methods priority in the UK [9].

Site selection for multicentre trials is done in a variety of ways but often relies on the investigators’ networks of colleagues or hospitals (often through NHS Research and Development departments in the UK) putting themselves forward. Not all of these sites will be suited to the trial’s recruitment task. The problem is distinguishing those that are from those that are not. There are some formal questionnaire-based methods [10, 11] but these are time-consuming and evidence that they are effective at selecting sites that will go on to recruit to target is lacking.

The Estimating Site Performance (ESP) project aims to see if it is possible to predict which sites will and will not meet their recruitment targets. Its approach has three components:

1. Minimise form-filling. We want to make use of the knowledge, experience and instincts of those tasked with setting-up sites: trial managers (TM).
2. Quantify the veracity of TMs’ knowledge, experience and instincts.
3. Suggest ways in which TMs’ knowledge, experience and instincts could be guided so as to improve predictions.

In short, can TMs predict which sites are worth investing energy in and should the trial team believe them?

## Methods

All TMs employed in the Centre for Healthcare Randomised Trials (CHaRT), Aberdeen, Scotland, UK, who were conducting site initiation visits (SIV) between 2014 and 2015, were invited to take part.

Participating TMs all received an information sheet about ESP and signed a consent form. Each TM was asked to predict the recruitment success of each site they opened after having completed the SIV. Predictions were collected on a form that comprised a ‘Yes/No’ answer to whether the site would recruit to target and a reason for the prediction (see Additional file 1). There was no guidance on the form as to the sorts of things that TMs might want to think about when making their prediction, rather it was a simple unguided ‘Why?’ question. The form also collected the number of years of trial management experience the TM had, together with details of the trial and recruitment targets for the site. TMs were invited to complete one form for each site opened. Prediction forms were completed on paper and placed in envelopes, which were sealed. The SIVs were timed around the time that local approval for the study was expected or given and before recruitment starting at the site. Limited feasibility work was carried out in participating trials in order to guide site selection and progression to SIV.

After a minimum of eight months of recruitment at each site for which a prediction had been made, all TMs in CHaRT were invited to a group discussion where predictions were presented together with sites’ actual recruitment performance over that period. Eight months was chosen largely on the pragmatic grounds that we considered it a suitable minimum period for sites to have overcome teething problems and to have established a good and steady recruitment process. Individual TMs reflected on their predictions and a general discussion about predicting sites’ recruitment performance and TMs’ ability and basis for predictions followed. The group discussion was audio recorded and transcribed verbatim.

### Analysis

Quantitative data from the prediction form were entered into IBM SPSS Statistics 25.0 for reporting of descriptives, percentages and frequencies. Site recruitment targets were recalculated to be *pro rata* for the duration of recruitment by the time of the group discussion meeting, meaning all predictions were judged against targets for the appropriate period. If the recruitment target was a range, e.g. 1–2 patients per month, the lowest number in the range was used. A site was deemed to have met its recruitment target if it met or exceeded that target.

Positive and negative predictive values (PPV and NPV, respectively), as well as sensitivity and specificity, were calculated for all TMs, TMs with < 2 years of experience and TMs with ≥ 2 years or more experience. Two years of experience was chosen arbitrarily as a reasonable cut-off point for the time it takes a TM to have acquired a range of relevant experience that allows him or her to judge when sites may struggle with recruitment. For our recruitment predictions, PPV and NPV, sensitivity and specificity can be defined as:

- *PPV*: what is the chance that a site predicted to hit its recruitment target will actually hit it?
- *NPV*: what is the chance that a site predicted to miss its recruitment target will actually miss it?
- *Sensitivity*: what proportion of sites that hit their recruitment target are identified as a ‘Yes, the site will recruit to target’ by TMs?
- *Specificity*: what proportion of sites that miss their recruitment target are identified as a ‘No, the site will not recruit to target’ by TMs?

Both the reasons for predictions given on the prediction forms themselves as well as the transcript of the group discussion were included in our qualitative analysis although this analysis focused more on the prediction forms than the group discussion. The reasons for the prediction provided on the prediction form were sorted into types of predictions according to the prediction made (Yes/No) and the accuracy of the prediction (Correct/Incorrect).

We wanted to develop a guided ‘*Will this site recruit to target?*’ question or questions: in other words, ‘*Will this site recruit to target? Think about x, y and z when making your prediction*’. Our starting point was that TMs’ unguided predictions might be adequate but that guided ones may be better; the problem was what guidance (the x, y and z) to give. The way this guidance would be operationalised led us to think about what might undermine recruitment success; in other words, ‘red flags’ that, if present, raise doubts about a site’s ability to recruit. Conventional content analysis [12] was therefore done on correct negative predictions (the site will not recruit to target) with the aim of identifying red flags that triggered the correct negative prediction. We also looked for clear signs of where the absence of a flag supported recruitment success, especially from the group discussion. This analysis was carried out *post hoc*.

## Results

All 10 eligible TMs took part and made predictions for 56 site visits relating to eight trials. One additional prediction form was completed that provided reasons for the prediction but did not record the actual prediction itself. This prediction form is therefore not included in our quantitative analysis. Thirty-nine of the predictions were made in connection with seven Phase III pragmatic randomised trials recruiting adults. The SIVs were mainly conducted face-to-face. The remaining 17 predictions came from a single non-randomised study, which used a launch meeting with a combination of other modes of SIVs (see Table 1). For this study, the two TMs divided the 17 sites between them for predictions (6 and 11 sites, respectively). For the randomised trials, two TMs attended three of the same SIVs and made separate predictions for the same sites. In all cases, predictions were made independently and without conferring with the other TM. Table 1 summarises the types of SIV by trial.

**Table 1:** Type of trial, associated trial managers (TM) and the type of site initiation visits (SIV) used

Trial ID	Type of trial	TMs making predictions (n)	Type of SIV				
			Face-to-face	Teleconference	Teleconference, launch meeting and other	Face-to-face, teleconference and launch meeting	Total SIVs (n)
1	Non-RCT	2	0	0	13	4	17
2	RCT	1	1	0	0	0	1
3	RCT	1	3	0	0	0	3
4	RCT	1	6	1	0	0	7
5	RCT	1	3	1	0	0	4
6	RCT	2	15	0	0	0	15^a^
7	RCT	1	1	3	0	0	4
8	RCT	1	5	0	0	0	5

^a^This number includes one visit where a prediction form was completed but the actual prediction was not recorded

*RCT* randomised controlled trial

For the group discussion, nine of the 10 TMs who had made predictions attended; one was unable to. An additional three TMs who had not made any predictions also attended. Only quotes from TMs who made predications have been used here.

### Predictions

The TMs’ predictions are presented in Tables 2, 3 and 4. Out of the 56 predictions made, 35 (62%) were correct and 21 (38%) were incorrect. From Tables 2, 3 and 4 it is clear that most predictions were ‘Yes’ (43/56, or 77%) and that most of these predictions were correct (the 65% PPV). TMs identified 82% (28/34) – their sensitivity – of all sites that hit their targets.

**Table 2:** Predictions made by all trial managers

	Actual Yes (met target)	Actual No (missed target)	Total
Predicted Yes	28	15	43
Predicted No	6	7	13
Total	34	22	56
Positive predictive value (%)	65	Negative predictive value (%)	54
Sensitivity (%)	82	Specificity (%)	32

**Table 3:** Predictions made by trial managers with < 2 years of experience

	Actual Yes (met target)	Actual No (missed target)	Total
Predicted Yes	7	8	15
Predicted No	1	2	3
Total	8	10	18
Positive predictive value (%)	47	Negative predictive value (%)	67
Sensitivity (%)	88	Specificity (%)	20

**Table 4:** Predictions made by trial managers with ≥ 2 years or more experience

	Actual Yes (met target)	Actual No (missed target)	Total
Predicted Yes	21	7	28
Predicted No	5	5	10
Total	26	12	38
Positive predictive value (%)	75	Negative predictive value (%)	50
Sensitivity (%)	81	Specificity (%)	42

There were fewer ‘No’ predictions (13/56, or 23%) and seven of these were correct (the 54% NPV). TMs identified 32% (7/22) – their specificity – of all sites that missed their targets.

Experience made a difference for the PPV (the chance a predicted ‘Yes’ is an actual ‘Yes’) and specificity (the proportion of sites that did not recruit to target that were correctly identified), which were both substantially higher in the more experienced group of TMs, though numbers in these subgroups were small. The NPV got worse with experience although, again, numbers were very small.

### Exploration of the written justification for predictions

The content analysis of written predictions identified eight distinct red flags linked to correct predictions of a failure to recruit to target (Table 5). The red flags identified in these predictions are described below in no particular order. They are numbered to guide the discussion of the flags below.

1. Previous poor performance

**Table 5:** An overview of the eight red flags identified in trial managers’ correct predictions of a failure to recruit to target

	Red flag
1	Previous poor performance
2	Slow/non-standard approval process
3	Patient or staff preferences or beliefs
4	Target for recruitment
5	Problems with the trial protocol and/or its implementation
6	Lack of engagement of site team
7	Lack of research experience of site staff/staff changes
8	Busy site staff

Some TMs had either personal experience of working with a site on other studies or the knowledge of site performance on other studies was shared within the Trials Unit. Knowing a site has previously performed poorly led to the expectation that this will happen on future trials.

‘*Previous record for recruiting for previous CHaRT trials has not been high (same PI).*’ *(*TM-5, < 2 years of experience, correct prediction of recruitment failure).

‘*Previous experience with site on a study, they only recruited one participant.*’ (TM-6, ≥ 2 years of experience, correct prediction of recruitment failure).

2. Slow/non-standard approval process

Although the overarching structure for approvals has been streamlined in the UK, the approval process can still vary considerably between individual sites as observed by one TM here. If the approvals stage is slow, it will eat into the time allocated to recruitment in the trial.

‘*Prolonged R&D approval process with lots of people required to sign off the study – very bureaucratic.*’ (TM-1, ≥ 2 years of experience, correct prediction of recruitment failure).

Reflections from the post-results group discussion also highlighted that the way the approvals process in the UK is timed and the way its ‘clocks’ start and stop can still cause delays for a trial even if it does not formally show up as approvals delay; the overall effect is still a delay to recruitment start.

‘*One site that just refused to take the document set for two months because they had R&D issues and staffing issues and they said, “We can’t do anything with this but we are timed on this so we don’t want it don’t give it to us”.*’ (TM-5 < 2 years of experience).

3. Patient or staff preferences or beliefs

One TM was told by a principal investigator (PI) that patients had a clear preference for a certain treatment. If most patients do not want to be randomised, this significantly limits the pool of potential participants available to a trial at that site.

‘*Appears, following discussion with PI at end of SIV [site initiation visit], that a lot of patients favour [treatment A over treatment B]…*’ (TM-5, < 2 years of experience, correct prediction of recruitment failure).

Reflections from the post-results group discussion also highlighted a lack of equipoise as a possible reason for recruitment failure:

‘*… and we were about halfway into it [the site visit], it was just obvious he was not in equipoise, he was not going to recruit to this trial… and lo and behold they closed down about six months later.*’ (TM-4, < 2 years of experience).

4. Target for recruitment

How recruitment targets are decided varies between trials and sites. Although a recruitment target is just a number, that number has significance in that it will affect motivation to recruit, especially if it is set unrealistically high at the outset. Here, the research nurse expressed doubt that the recruitment target for the site was realistic. This realisation is demoralising to the research nurse and likely to impact on their efforts to recruit.

‘*Research nurse…was doubtful about target recruitment.*’ (TM-6, ≥ 2 years of experience, correct prediction of recruitment failure).

Reflections from the post-results group discussion also highlighted that recruitment targets do not get much attention at the early stages, are set artificially and for some trials appear to have been set too low across sites.

‘*Well with hindsight looking at the targets that are set they were too low*.’ (TM-9, ≥ 2 years of experience).

‘[…site name…] *they done really well but did promise a lot more. So their target was small, they did promise that they had, you know, hundreds of people that they could contact. So I mean although they did meet prediction and they did recruit really, really well I still would’ve expected more from them*.’ (TM-8, < 2 years of experience, reflecting on a correct prediction of recruitment success).

Combining an incentive with the ‘per patient’ payment is, however, one way of encouraging sites to reach their target.

‘*…and [site] had an incentive because we said if they got to 20 we’d pay for them to have a [name of equipment], which is a piece of equipment they needed for the study and we said if they didn’t reach 20 they’d have to make a contribution to that piece of equipment…And they reached 20 and stopped*.’ (TM-7, ≥ 2 years of experience, reflecting on a correct prediction of recruitment success).

5. Problems with the trial protocol and/or its implementation

The nature of a trial can make it more or less difficult to recruit to across sites. Issues with the trial protocol are likely to be generic across all sites, although the degree to which it affects recruitment may vary depending on a site’s capacity to work around the challenges the protocol presents, particularly if this is at odds with the local patient pathways. Here, a research nurse recognised the trial as difficult to recruit to at the SIV. Trials that are more difficult to recruit to place a higher demand on the site team and this influences their engagement with the trial.

‘*RN [research nurse] did recognise it was a difficult trial to recruit to…*’ (TM-6, ≥ 2 years of experience, correct prediction of recruitment failure).

Reflections from the post-results group discussion also highlighted that a mismatch between the local care pathway and the trial design can cause an issue:

‘*And they just said “This is just not how things work here, it’s just impossible to execute.” So you find out these little gems when the PI isn’t around.*’ (TM-2, ≥ 2 years of experience).

Sometimes, the way a site is physically set up, e.g. split so that there are several hospitals that can recruit to the trial but they are only counted as one site, can cause delays in set-up, especially if the TM is only made aware of this at the SIV. Here, the TM notes that due to the site being split there would be two pharmacies involved, which has implications for the set-up process and can cause delays to recruitment start.

‘*Split site, two pharmacists involved.*’ (TM-7, ≥ 2 years of experience, correct prediction of recruitment failure).

Reflections from the post-results discussion also highlighted site facilities as a reason for recruitment failure:

‘*Like in [name of site]…, it turned out that they have a massive [name of treatment] centre and they have no surgery spaces for [name of alternative treatment] so how can then they offer ... how can they randomise to have the treatments and say that they’ll get the treatments in eight weeks ... because they don’t have a surgery space.*’ (TM-6, ≥ 2 years of experience, reflecting on correctly predicting recruitment failure).

An important function of the SIV is often that the whole team is gathered and there is an opportunity to plan how recruitment will work and distribute responsibility for all the tasks involved. However, this process works best if the parties involved have come prepared. If the way recruitment is going to be done cannot be agreed early, it is likely to cause delays to recruitment start.

‘*Lots of confused discussion between team about how best to identify patients and recruit them.*’ (TM-7, ≥ 2 years of experience, correct prediction of recruitment failure).

‘*Lots of problems and difficulties thrown at me at SIV teleconference.*’ (TM-1, ≥ 2 years of experience, correct prediction of recruitment failure).

Whereas for successfully recruiting sites, this was one of the main purposes of the SIV.

‘…*Thought about recruitment in advance and have identified PICs [Participant Identification Centres]* …’ (TM-7, ≥ 2 years of experience, reflecting on correct prediction of recruitment success).

‘*The site have proactively pushed for teleconference to arrange site training – were very engaged at the teleconference and asked questions about approaching patients. They had identified potential participants at PI clinic before the teleconference*…’ (TM-9, ≥ 2 years of experience, reflecting on correct prediction of recruitment success).

6. Lack of engagement of site team

The level of engagement of a site with the trial will be evident to the TM throughout the set-up process and usually before the SIV. Often the PI is essential to recruitment of a trial as he or she will often be the person who first introduces patients to the trial, which may not happen if the PI is not fully engaged with the trial.

‘*PI only engaged in the study at a very late stage*.’ (TM-1, ≥ 2 years of experience, correct prediction of recruitment failure).

Reflections from the post-result group discussion also highlighted that a PI’s engagement can vary over the lifespan of the trial, highlighting that this is an issue throughout.

‘*[Site name], although recently the research nurse has advised that she’s having issues with engagement from the PI there. Again, looking back at the site initiation as well there were some signs there, but again, initially when they started recruiting they were recruiting quite well and it’s just been the last few months that it’s sort of slowed down*… *Lack of engagement with the PI, he’s just stopped… there’s lack of communication with the research nurse*.’ (TM-5, < 2 years of experience, reflecting on an incorrect prediction of recruitment success).

The level of engagement of site team members can be picked up in many ways, even body language at the SIV.

‘*PI listened but seemed keen to have SIV over.*’ (TM-10, ≥ 2 years of experience, correct prediction of recruitment failure).

Conversely, good engagement supports recruitment:

‘*…PI has sent emails to R&D on her own initiative to chase up R&D approval to allow site opening. …*’ (TM-9, ≥ 2 years of experience, correct prediction of recruitment success).

‘*Very engaged team who have discussed early staffing issues with us up-front and plan to work around this in the long term.*’ (TM-1, ≥ 2 years of experience, correct prediction of recruitment success).

Reflections from the post-results discussion also illustrate how this kind of lack of engagement is a warning sign of likely recruitment failure:

‘*The PI was quite awkward I felt ... how did it work if he saw a patient out in rural hospital, would they have to come into the main hospital, how would the research nurses know he’s seen a patient ... just lots and lots of ifs and buts that I kind of should’ve flagged up more warning signs at the start I think.*’ (TM-7, ≥ 2 years of experience, reflecting on an incorrect prediction of recruitment success).

There is a lot of background work involved in planning and conducting SIVs; often many site team members will be invited as it is important that they receive the training relevant to the trial. If a member of the site team does not attend the SIV, alternative arrangements will have to be made, which again is likely to slow down the trial progress at the site.

‘*Other named consultant who will recruit did not attend SIV*.’ (TM-10, ≥ 2 years of experience, correct prediction of recruitment failure).

Reflections from the post-results discussion also highlighted lack of engagement with the SIV as a factor affecting recruitment success:

‘*They’re just… I mean I said lots of problem… at their site initiation visit there was just… the PI never came to the investigator meeting, the site visit was a nightmare to even organise.*’ (TM-1, ≥ 2 years of experience, reflecting on correctly predicting recruitment failure).

‘*…but just half an hour of dedicated time for the meeting would be good. … I don’t know whether really it’s made any difference me coming here because I don’t think they were listening. … And then when people don’t attend, that’s the worst as well.*’ (TM-2, ≥ 2 years of experience).

Throughout the lifespan of a trial there needs to be a flow of communication between the trial office and sites and if this flow is poor, then this will slow down the trial at that site.

‘*Email communication is slow.*’ (TM-10, ≥ 2 years of experience, correct prediction of recruitment failure).

Reflections from the post-results discussion also highlighted this as a reason for recruitment failure:

‘*[name of site] were always, they were just a bit cagey.*’ (TM-10, ≥ 2 years of experience, reflecting on correctly predicting recruitment failure).

Conversely, good communication supports recruitment:

'*And I know in [trial] you know, the sites that have probably done really, really good are the ones that we’ve had that great communication with, the ones that picked up the phone whenever there’s been the slightest query and we’ve been able to deal with that really quickly and then away we’ve went again… But I think yeah, to keep that communication going is probably one of the key things*.’ (TM-7, ≥ 2 years of experience).

7. Lack of research experience of site staff and staff changes

The lack of research experience of staff, for both PIs and research nurses, was noted as a problem. Having site staff without research experience is also likely to slow down the trial at a site due to research-naïve staff having to become familiar with the research process in general as well as with the procedures involved for specific trials.

‘*PI first time as PI; two other consultants relatively research naïve.*’ (TM-7, ≥ 2 years of experience, correct prediction of recruitment failure).

‘*The site is about to lose an experienced research nurse and promote a nurse with no research experience.*’ (TM-10, ≥ 2 years of experience, correct prediction of recruitment failure).

Reflections from the post-results discussion also highlighted turnover of site staff as a factor affecting recruitment success:

‘*[name of site] I think have had quite a few changes of staff so that might explain quite a lot of those issues.*’ (TM-4, < 2 years of experience, reflecting on an incorrect prediction of recruitment success).

Although the discussion also highlighted that there are exceptions.

‘*Well yeah, there’s almost a complete turnover in people at [name of site], but they have been good. The new guy’s brilliant … Well yeah, they’ve got, well everyone who was at the site visit is no longer there.*’ (TM-10, ≥ 2 years of experience, reflecting on an incorrect prediction of recruitment failure).

8. Busy site staff

Often a site is involved in multiple trials and if a TM knows the site staff have a high team workload there is an anticipation that their trial may not be prioritised by busy site staff.

‘*[name of research nurse] but she has many studies to deal with.*’ (TM-10, ≥ 2 years of experience, correct prediction of recruitment failure).

Reflections from the post-results group discussion also highlighted this as a reason for recruitment failure:

‘*Well looking back now at the site initiation the PI was very busy, she had just come off a nightshift and she was wanting to do everything. So thinking back now in retrospect there was warning signs then.*’ (TM-5, < 2 years of experience, reflecting on an incorrect prediction of recruitment success).

‘*…and they have no support currently at all. They’re taking the projects on because they want them on the books, but they have absolutely no network to support it…*’ (TM-2, ≥ 2 years of experience).

## Discussion

Trials are hard work and we want that work to be worth it. Investing substantial amounts of work in trial sites that fail to recruit is something to try and avoid because there is plenty of work to do elsewhere in the trial. Some work has been done with site selection questionnaires [10, 11] but we wanted to see if something simpler was both possible and useful with regard to likely recruitment success: asking the TMs setting up the site what they thought.

We asked TMs to make simple Yes/No predictions and we gave no guidance as to what they should think about when making their predictions. Ten TMs made 56 predictions across eight trials and it turns out that TMs are pretty good at making these predictions. If we think of TMs as a diagnostic test of recruitment, our TMs had a sensitivity of 82% and a specificity of 32%, correctly identifying 65% of sites that would hit their recruitment target and 54% of those that did not.

The 32% specificity is the critical feature of this diagnostic test and it is very low. As mentioned in the ‘Analysis’ section, we were particularly interested in sites that were predicted to be poor recruiters because these consume a substantial amount of trial management time and resource for little return. A specificity of 32% is too low to take decisive action based on a poor recruitment prediction. It seems premature, for example, to recommend rejection of sites predicted to be poor recruiters or stopping the support given to them.

We expected this and the work described here was always intended as the first stage of our ESP work. This is why we also looked for ‘red flags’—factors associated with recruitment failure—because we suggest that these can be used to provide guidance to TMs when thinking about their recruitment predictions. Table 5 shows the eight red flags we identified in our qualitative work. We anticipate that the more red flags that are identified during site-set-up, the more likely it will be that a TM would be justified in making a ‘No’ prediction.

It is clear that there is some overlap between flags and that some may well be more important than others. We do not think the flags should be a simple box-ticking exercise but be used as a prompt for discussion when doing a SIV. Discussion of the flags after the visit by the central trial team could support decisions about which sites will need more support, which less and whether poor recruitment expectations at some sites means new sites are needed. Some could also be considered before the SIV. In particular, previous poor performance, the recruitment target, problems with the trial protocol and/or its implementation and lack of research experience of site staff/staff changes could be considered early on in site identification and set-up. We did not use the predictions to influence trial conduct in the work described in this paper, but it is easy to imagine that ticks against these red flags well before the SIV could give the central trial team reason to reconsider whether the site was worth pursuing. At the very least, the site might fall down the order list of which sites are brought on-stream or perhaps a member of the team could do a ‘site selection visit’ (in person or by teleconference) to confirm whether it was worth moving ahead with the site’s involvement in the trial. We also think that the flags might be a helpful training tool for new or inexperienced TMs to provide a basic structure to the discussions they have with sites during set-up and at SIVs.

By the end of the SIV, it may be possible to make a judgement about recruitment success by looking across the red flags, combined with any other relevant information the TM has. The TM could create a matrix listing all sites and their red flags, which would give an overview of high-risk sites, those not expected to recruit to target. The aim, of course, would be to have mostly low-risk sites, with very high-risk sites having been excluded before the SIV or put on hold until one or more red flags change. That matrix will give the central trial team an indication of where their limited resources should be targeted when thinking of sites and recruitment.

### Strengths and limitations

There are a number of limitations. First, the work was done at a single trials unit and involved a relatively small number of TMs. While clearly a limitation, this work was the first stage of the project so its limited scale seems appropriate. Moreover, we expected predictions to be improved by having red flags and identifying those flags was part of this work. Now we have those red flags, further work needs to be on a bigger scale and involve more trials units and teams. To this end, we have developed a revised prediction form (see Additional file 2) and plan to test this further both among TMs based in CHaRT and across the UK; we ask that TMs who are interested in joining an evaluation of the new prediction tool get in touch with us. The second limitation was that the predictions were made after the SIV by which point much of the TM’s work has already been invested in the site regardless of the prediction. Again, while true we (1) had no intention of acting on the predictions because we had no idea how good they were and (2) we wanted the red flags to be as well-informed as they could be. After the SIV therefore seemed the best point at which to make the prediction. As mentioned earlier in the ‘Discussion’ section, some of our red flags could easily be considered before the SIV and trial teams could in principal take action based on their assessments. The third limitation was that most predictions were made for face-to-face site initiation visits. It can be argued that it is easier to build good rapport with sites staff at face-to-face meetings and there is obviously an opportunity to pick up on body language cues too. However, few prediction justifications were based on body language rather than actual behaviour and we did not see signs of behaviour change (e.g. differences in attendance) due to the mode of the visit, though a larger study may have seen a difference. Face-to-face SIVs are expensive in time and money and other modes might be preferred. Giving TMs red flags to consider, some before the SIV, may allow decisions about when to use a face-to-face meeting and when to limit investments in sites by using other modes..

We think the study also has some strengths. It set out with the intention of tapping into the experience of TMs rather than to ask site or trial staff to do substantial amounts of form-filling. Even in the complete absence of guidance, a very simple form and TMs’ experience led to a reasonable first shot at predictions. The qualitative work, especially the post-results reflection, leave us reassured that the red flags have face-validity. For example, they map nicely onto the issues listed on the Clinical Trials Toolkit, a tool to provide practical advice to researchers in designing and conducting publicly funded clinical trials in the UK (http://www.ct-toolkit.ac.uk/routemap/feasibility-and-investigator-selection/). We are confident that any TM looking at the list in Table 5 will nod his or her head in weary agreement. Finally, the work was good fun, captured recruitment intelligence from > 50 site initiation visits and led to a modified but still simple tool that we plan to test and all without creating a mass of extra work for TMs.

## Conclusions

Poorly recruiting sites soak up considerable time and resource in return for a handful of participants. It would be best for everyone and particularly central trial teams if we could predict which sites these would be and either fix things or wave a polite goodbye.

In this small study, we asked 10 TMs to make simple, unguided Yes/No predictions about site recruitment in eight trials; they correctly identified 82% of sites that did hit their recruitment target and 32% of those that did not. Crucially, the latter—correctly identifying sites that will fail to recruit—is not good enough for decision-making. However, the study also provided us with a list of eight red flags—factors that are linked to poor recruitment—which we think will make TMs’ future predictions better.

We have developed a modified prediction form including these red flags and encourage anyone interested in contributing to its evaluation to contact us.

## Additional files

Additional file 1: ESP prediction form. The form used to collect TMs’ predictions in the current study. (DOC 39 kb)

Additional file 2: Modified prediction form. The modified prediction form for the evaluation of ‘red flags’ identified in the current study. (DOCX 29 kb)

Additional file 3: Quantitative data. (XLS 18 kb)

## Abbreviations

CHaRT: The Centre for Healthcare Randomised Trials
CI: Chief Investigator
PI: Principal investigator
PIC: Participant Identification Centre
RN: Research nurse
SIV: Site Initiation Visit
TM: Trial manager
UK: United Kingdom
