# Supplementary material for: Estimating Site Performance (ESP): can trial managers predict recruitment success at trial sites? An exploratory study
Source: Trials. 2019 Apr 3;20:192. doi: 10.1186/s13063-019-3287-6 (PMC6448211; doi:10.1186/s13063-019-3287-6)
Supplement: Supplementary file 1 — ESP prediction form. The form used to collect TMs’ predictions in the current study. (DOC 39 kb) [file 13063_2019_3287_MOESM1_ESM.doc]

**ESP recruitment prediction form**

| Name of trial manager |  | | | Name of trial | | |  | |
| --- | --- | --- | --- | --- | --- | --- | --- | --- |
| How long have you been a trial manager? | | |  | What CTU are you based at? | |  | | |
| Site visiting | |  | | Date of prediction | |  | | |
|  | | | | | | | | |
| What is the site’s recruitment target? | | |  | | | | | |
| Which of the following as the site had?  *Circle as many as necessary* | | | On site SIV | Teleconf SIV | Launch meeting | | | Other |
| What is the site status? | | | Opened for recruitment | | Abandoned | | | |
|  | | | | | | | | |
| In your opinion, will this site recruit to its target on time? | | | Yes | | No | | | |
| Why do you think this (for sites being opened)?  **OR**  Why was the site abandoned?  *Please continue overleaf if needed* | | |  | | | | | |
| Are there any other issues with the site?  *Please continue overleaf if needed* | | |  | | | | | |
