# Supplementary material for: Estimating Site Performance (ESP): can trial managers predict recruitment success at trial sites? An exploratory study
Source: Trials. 2019 Apr 3;20:192. doi: 10.1186/s13063-019-3287-6 (PMC6448211; doi:10.1186/s13063-019-3287-6)
Supplement: Supplementary file 2 — Modified prediction form. The modified prediction form for the evaluation of ‘red flags’ identified in the current study. (DOCX 29 kb) [file 13063_2019_3287_MOESM2_ESM.docx]

| **ESP 2 – PREDICTION FORM -** To be completed shortly after the Site Initiation Visit (SIV) | | | | | | | | | | | | | | | | | | | | | | | | | | | | | | | | | | | | | | | | | | | | | | | |
| --- | --- | --- | --- | --- | --- | --- | --- | --- | --- | --- | --- | --- | --- | --- | --- | --- | --- | --- | --- | --- | --- | --- | --- | --- | --- | --- | --- | --- | --- | --- | --- | --- | --- | --- | --- | --- | --- | --- | --- | --- | --- | --- | --- | --- | --- | --- | --- |
| **TM ID** |  | | | | | | | | | | | | **Trial ID** | | | |  | | | | | | | | | | | | | | | | | **Site ID** | | |  | | | | | | | | | | |
|  | | | | | | | | | | | | | | | | | | | | | | | | | | | | | | | | | | | | | | | | | | | | | | | |
| **Date of SIV** | | **D** | **D** | **\** | **M** | **M** | **\** | **Y** | **Y** | **Y** | **Y** | **Date of prediction** | | | | | **D** | | | **D** | **\** | **M** | **M** | **\** | | **Y** | **Y** | **Y** | | **Y** | **Date opened to recruitment  (‘green-lighted’)** | | | | | | | **D** | **D** | **\** | **M** | **M** | **\** | **Y** | **Y** | **Y** | **Y** |
|  | | | | | | | | | | | | | | | | | | | | | | | | | | | | | | | | | | | | | | | | | | | | | | | |
| **The site’s recruitment target for the duration of the  recruitment period** | | | | | | | | | | | | |  | | | | | | **The type of SIV the site had (face-to-face at site, teleconference, launch meeting, other)** | | | | | | | | | | | | | | | | |  | | | | | | | | | | | |
|  | | | | | | | | | | | | | | | | | | | | | | | | | | | | | | | | | | | | | | | | | | | | | | | |
| **Before deciding whether you think this site will recruit to its target and on time, please consider the following for the site and tick accordingly.  Use the comments/notes section to keep track of the information you have collected and/or observed about the site.** | | | | | | | | | | | | | | | | | | | | | | | | | | | | | | | | | | | | | | | | | | | | | | | |
| **‘Flag’** | | | | | | | | | | | | | | | | | | | | | | | | | **Yes** | | | | **No** | | | **Don’t know** | **Comment/notes** | | | | | | | | | | | | | | |
| **1. Has the site previously recruited well for a similar trial (same disease area)?  Think about:** Has anything changed since then? | | | | | | | | | | | | | | | | | | | | | | | | | 🞏 | | | | 🞏 | | | 🞏 |  | | | | | | | | | | | | | | |
|  | | | | | | | | | | | | | | | | | | | | | | | | |  | | | |  | | |  |  | | | | | | | | | | | | | | |
| **2. Has the approval process run smoothly for this this site?** | | | | | | | | | | | | | | | | | | | | | | | | | 🞏 | | | | 🞏 | | | 🞏 |  | | | | | | | | | | | | | | |
|  | | | | | | | | | | | | | | | | | | | | | | | | |  | | | |  | | |  |  | | | | | | | | | | | | | | |
| **3. Are the site staff in equipoise for the trial intervention?**  **Think about:** is there any indication that patients have a treatment preference? | | | | | | | | | | | | | | | | | | | | | | | | | 🞏 | | | | 🞏 | | | 🞏 |  | | | | | | | | | | | | | | |
|  | | | | | | | | | | | | | | | | | | | | | | | | |  | | | |  | | |  |  | | | | | | | | | | | | | | |
| **4. Are site staff confident the site can reach the recruitment target?** | | | | | | | | | | | | | | | | | | | | | | | | | 🞏 | | | | 🞏 | | | 🞏 |  | | | | | | | | | | | | | | |
|  | | | | | | | | | | | | | | | | | | | | | | | | |  | | | |  | | |  |  | | | | | | | | | | | | | | |
| **5. Did the site staff think the trial would be easy? Think about:** *the trial protocol; have the site team thought about/prepared a recruitment strategy?* *Were there any unexpected site issues such as split sites?* | | | | | | | | | | | | | | | | | | | | | | | | | 🞏 | | | | 🞏 | | | 🞏 |  | | | | | | | | | | | | | | |
|  | | | | | | | | | | | | | | | | | | | | | | | | |  | | | |  | | |  |  | | | | | | | | | | | | | | |
| **6. Is the site team engaged? *Think about:*** *Is the PI engaged?* *How was attendance at the SIV?* *How is communication between trial office and site?* | | | | | | | | | | | | | | | | | | | | | | | | | 🞏 | | | | 🞏 | | | 🞏 |  | | | | | | | | | | | | | | |
|  | | | | | | | | | | | | | | | | | | | | | | | | |  | | | |  | | |  |  | | | | | | | | | | | | | | |
| **7. Do site staff have research experience? *Think about:*** *What is the previous research experience of the site team?* *Are there any known staff changes coming up? If yes, are there any plans regarding continuing work on the trial?* | | | | | | | | | | | | | | | | | | | | | | | | | 🞏 | | | | 🞏 | | | 🞏 |  | | | | | | | | | | | | | | |
|  | | | | | | | | | | | | | | | | | | | | | | | | |  | | | |  | | |  |  | | | | | | | | | | | | | | |
| **8. Do site staff appear well organised? *Think about:*** *Are staff working on many other studies?* *Is the department recruiting to other competing trials?*  *Are any finishing soon?* | | | | | | | | | | | | | | | | | | | | | | | | | 🞏 | | | | 🞏 | | | 🞏 |  | | | | | | | | | | | | | | |
|  | | | | | | | | | | | | | | | | | | | | | | | | | | | | | | | | | | | | | | | | | | | | | | | |
| **Any other comments on the site or observations made at the SIV? Please describe.** | | | | | | | | | | | | | | | | | | | | | | | | | | | | | | | | | | | | | | | | | | | | | | | |
| **In your opinion, will this site recruit to target on time?** | | | | | | | | | | | | | | Yes | | 🞏 | | No | | | | | 🞏 | | |  | | | | | | | | | | | | | | | | | | | | | |
| **Which, if any, of the information above has influenced your prediction the most? Please rank up to three ‘flags’ that influenced your prediction in order of importance, 1 being most important and contributed most to your prediction, 2 being less important and contributing less to your prediction etc. If there are other issues that influenced your prediction, please write these in the relevant boxes.** | | | | | | | | | | | | | | | | | | | | | | | | | | | | | | | | | | | | | | | | | | | | | | | |
| **1.** | | | | | | | | | | | | | | | **2.** | | | | | | | | | | | | | | | | | | | | **3.** | | | | | | | | | | | | |
